# Early life household intactness and timing of pubertal onset in girls: a prospective cohort study

**DOI:** 10.1186/s12887-020-02345-w

**Published:** 2020-10-28

**Authors:** Sara Aghaee, Julianna Deardorff, Louise C. Greenspan, Charles P. Quesenberry, Lawrence H. Kushi, Ai Kubo

**Affiliations:** 1 Kaiser Permanente Division of Research, 2000 Broadway, CA 94612 Oakland, USA; 2 Division of Maternal and Child Health, School of Public Health, University of California, 2121 Berkeley Way #5302, CA 94720 Berkeley, USA; 3 Kaiser Permanente San Francisco Medical Center, 2425 Geary Boulevard, CA 94115 San Francisco, USA

**Keywords:** Adolescent health, Developmental origin of health and diseases, Health disparities, Household structure, Puberty

## Abstract

**Background:**

Girls who experience early-life familial stress may have heightened risk of early puberty, which has adverse implications for adolescent and adult health. We assessed the association between household intactness and pubertal onset using a racially/ethnically diverse cohort of girls from Northern California.

**Methods:**

A prospective cohort study of 26,044 girls born in 2003-10. Girls living with both parents from birth up to 6 years were considered to come from “intact” households while others constituted “non-intact” households. Pubertal development was measured using pediatrician-assessed Tanner staging for breast and pubic hair. Pubertal onset was defined as the transition from Tanner Stage 1 to 2+ for breast (thelarche) and pubic hair (pubarche). Menarche data was collected from routine well-child questionnaires. Weibull regression models accommodating left, right, and interval censoring were used to determine risk of earlier thelarche and pubarche, and logistic regressions were used to assess the risk of early menarche (age < 12).

**Results:**

Girls exposed to non-intact households before age 2 years were at increased risk for earlier thelarche and pubarche with significant effect modification by race/ethnicity, compared with girls from intact households. The associations were strongest among Black girls (adjusted hazard ratio [HR]: 1.60, 95% confidence interval [CI]: 1.29,1.98; HR: 1.42, 95%CI: 1.15,1.77 for thelarche and pubarche, respectively). There were no significant associations among Asian/Pacific Islanders. Girls who lived in non-intact households before age 2 years were also at increased risk for earlier menarche, but without race/ethnic interaction. Adjustment for prepubertal obesity did not change these associations. Associations between living in non-intact households after age 2 years and early puberty were weaker but still significant.

**Conclusions:**

Exposure to a non-intact household early in life may increase the risk of early puberty in girls. Future psychosocial interventions focused on improving family cohesiveness and efforts to reduce childhood stress among families that are non-intact may mitigate these negative associations, thereby preventing future adverse health effects of early puberty and health disparities.

## Background

Girls in the United States are experiencing puberty earlier compared with just a few decades ago [1]. This trend has been recognized as an important public health issue due to the higher risk of mental, emotional, and physical health conditions associated with earlier puberty. These include depression, eating disorders, substance use, risky sexual activities, early sexual debut, and underage pregnancy [2–4], in addition to later-life reproductive cancers and cardiovascular disease [2, 5]. Race/ethnic disparities in pubertal outcomes present further cause for concern. One stark example of this can be seen in the difference in age at onset of breast development (thelarche). We have reported that the median age of thelarche is about 8.8 years in Black girls and 9.3 in Hispanic & Latinx girls, compared to 9.7 in Whites [1]. Understanding the predictors driving this decline in age of pubertal onset and causes of racial/ethnic differences is crucial to improving the health of race/ethnic minorities in the United States, a population already negatively affected by health disparities.

Absence of a biological father has long been theorized to have an accelerating effect on girl’s reproductive development and may present one possible exposure by which race/ethnic minorities may be at a greater risk for developing earlier puberty. Ellis’ Child Development Theory argues that children gauge future reproductive opportunities based on present psychosocial exposures (i.e., father absence, lack of resources, etc.) and develop reproductive strategies that may delay or accelerate puberty to meet these expectations [6]. Findings from past research also suggest that children may be particularly sensitive to these exposures during the first few years of life [6]. Further, infant attachment, a phenomenon associated with pubertal timing and other developmental milestones, occurs within the first two years of life [7, 8]. Results from past studies, including ours, show that children raised without their biological father are more likely to undergo pubertal development earlier than children raised with both parents [9–15], lending support to this theory. However, these studies have several methodological limitations. First, family structure and household environment are determined using different measures, and timing of these factors span from early-childhood to adulthood making it difficult to establish temporality and to understand the role of the “sensitive-period” of learning. Second, many of these studies use self-reported measures of secondary sexual characteristics (i.e., breast and pubic hair) or use menarche as a proxy for pubertal onset despite menarche occurring much later than onset of secondary sexual characteristics. Lastly, despite the disproportionately high rates of single motherhood in Black communities [16], little is known regarding the impact of household intactness on pubertal trajectories among racial/ethnic minorities because the majority of previous studies were conducted using predominantly White cohorts. We addressed these limitations and reduced the gap in knowledge by examining race/ethnicity-specific associations between household intactness and onset of puberty, accounting for the age of exposure to a non-intact household environment (age < 2 vs. ≥ 2), using clinician-measured prospective data in a large, racially/ethnically diverse cohort of girls from Northern California.

## Methods

### Participants

This study was conducted using a prospective cohort of young girls within Kaiser Permanente Northern California (KPNC), a large integrated health care delivery system that serves over 4.4 million members in Northern California. KPNC electronic health record (EHR) and research databases were used to identify full-term (> 36 weeks gestation), singleton girls born at a KPNC medical facility between 2003 and 2010. Girls were required to have continuous KPNC membership (no coverage gap > 90 days) during the follow-up period, in order to minimize missing data. Girls were included if mothers did not have extreme pregravid body mass index (BMI) (< 15 kg/m^2^ or > 60 kg/m^2^), were ≥ 18 years at delivery, had at least one prepubertal BMI measurement and one Tanner Stage assessment (breast and/or pubic hair), and had documentation of household structure before age 2 years and anytime between ages 2 and up to 6 years. Girls with medical conditions affecting pubertal development, such as congenital adrenal hyperplasia or adrenal tumors, were excluded. The follow-up period for assessing pubertal development went through June 30, 2020.

Girls missing data on clinically important covariates (e.g., median household income) were excluded from analyses, resulting in a final analytical cohort of 26,044 girls. The study was conducted in accordance with prevailing ethical principles. All data were gathered from KPNC clinical and administrative databases following study approval and waiver of consent by the KPNC Institutional Review Board.

### Measurements

#### Exposure variable (household intactness)

“Well Baby/Child” visits are used by KPNC primary care providers to discuss and assess children’s health and overall wellbeing with parents/caregivers. These visits, which occur about 10 times during the first 2 years of the child’s life, then 3 times between ages 2–6 years, include general check-ups along with health questionnaires covering nutrition, safety, parenting behaviors, and family/household circumstances. In approximately half of these visits, parents/caregivers are asked if the child is currently living with both parents. We categorized household intactness into three mutually-exclusive categories as: “Intact household” if the response to this question was “Yes” at all timepoints from birth until age 6 years; “Non-intact household before age 2 years” if the response was “No” at any timepoint before age 2 years and either “Yes” or “No” between the ages of 2 and up to 6 years; “Non-intact household during age 2–6 years” if the response was “Yes” at all timepoints before the age of 2 years but “No” anytime between the ages of 2 and up to 6 years.

#### Outcomes

In 2010, KPNC providers began documenting Tanner Stages as part of routine pediatric checkups starting at age 6 years. Tanner Stage for pubic hair and breast development is assessed using established 5-level Tanner Stages by girls’ primary physician [17]. Breast development is determined using a combination of palpation and visual inspection, while pubic hair development is assessed using visual inspection only. Age at menarche was estimated using responses to well-child or well-teen (for girls age 13+) questionnaires (“Has your daughter started menstruating?” or “Have you started your period?”). Girls who indicated having gotten their menses before age 12 years were classified as ‘early’ menarche [18], while girls who denied having gotten their menses before or after age 12 years were considered to have normal/late menarche. Girls may choose to opt-out of this screening and primary care providers often do not document Tanner Stage when full maturation is apparent. For these reasons, not all KPNC youth have information on Tanner staging documented in their medical record.

We have confirmed the validity of using KPNC EHR Tanner data in our previous study, the Cohort Study of Young Girls’ Nutrition, Environment, and Transitions (CYGNET) [19]. In CYGNET, research staff were rigorously trained to Tanner Stage a cohort of 444 girls over a span of 10 + years. The quality of Tanner staging was frequently assessed by a Kaiser pediatric endocrinologist (LCG). We compared Tanner assessments done by research staff with those done by KPNC providers within six months of each other (*n* = 217) and found weighted kappas of 0.66 (95%CI: 0.61, 0.72) for breast and 0.65 (95%CI: 0.59, 0.71) for pubic hair (unpublished data). Since then, we have conducted further validation analyses by measuring Tanner agreement in girls who were assessed by two different clinicians within six months of each other. In over 300 girls, the weighted kappas were 0.73 (95%CI: 0.69, 0.77) for breast and 0.72 (95%CI: 0.68, 0.76) for pubic hair. Among overweight/obese girls weighted kappas were 0.68 (95%CI: 0.53, 0.83) and 0.68 (95%CI: 0.54, 0.83), respectively.

In this study, our primary outcomes of interest were age at transition from Tanner Stage 1 (prepubertal) to Tanner Stage 2+ for breast (thelarche) and pubic hair (pubarche) development.

#### Covariates, Mediator, Effect Modifier

Girl’s prepubertal weight and height were obtained from clinic visits in the time window within 60 days prior to or after the last known Tanner Stage 1 assessment. BMI percentiles were calculated using age- and sex-specific Centers for Disease Control and Prevention (CDC) year 2000 standard population distributions [20].

Data on girl’s race/ethnicity (White, Black, Hispanic & Latinx, Asian/Pacific Islander, and Other/Unknown) and median household income were also obtained from KPNC EHR. Median household income was determined using 2010 Census data for girls’ residential address at time of birth. Residential addresses within a year of birth were used for girls who did not have documented residential information at birth. About 97% of girls had home addresses on file, while the remaining 3% had only zip code or city name.

### Statistical analyses

The association between household intactness and pubertal onset was determined using parametric survival (Weibull) regression models, which account for left, right, and interval censoring [21]. Girls were considered left-censored if they had already transitioned to Tanner Stage 2+ at the time of the first Tanner Stage exam (baseline) and right-censored at the time of their last exam if they had not transitioned to Tanner Stage 2+ by the time of the last exam (end of follow-up) or had only 1 assessment at Tanner Stage 1. Girls were considered interval-censored if they had an exam with assessments at Tanner 1 with a later exam at Tanner Stage 2+, with the exact age of transition between the assessed ages at the two stages being unknown. The models produce two effect size measurements: the time ratio (TR) and the hazard ratio (HR). TR estimates represent the ratio of the median time to event for a given level of the exposure variable in relation to its reference level. We also explored associations between household intactness and menarche as a secondary outcome, using binary logistic regression models (reference= ‘menarche ≥ 12 years old’). Girl’s race/ethnicity and median household income were included in all multivariate models as potential confounders.

We examined race/ethnicity as a potential effect modifier by using a cross-product term of girl’s race/ethnicity and household intactness. The test for interaction was statistically significant in thelarche and pubarche models (*p* < 0.05). As a secondary analysis, we explored the mediating role of girl’s prepubertal BMI (percentiles) by comparing the effect estimates of the association between household intactness and each pubertal outcome before and after inclusion of this variable in the model. All analyses were conducted using SAS version 9.4 (SAS Institute, Cary, NC).

## Results

### Participant characteristics

Table 1 presents demographic characteristics of the study participants. About 84% (*n* = 21,824) of girls lived in intact households between birth and up to age 6 years, while 8% (*n* = 2,034) lived in non-intact households before the age of 2 years and 8% (*n* = 2,186) between the ages of 2 and 6 years. About 64% of the study population was non-White (7% Black, 26% Hispanic & Latinx, 22% Asian/Pacific Islander, and 8% Other/Unknown). Almost half of Black girls lived in non-intact households before age 6, the highest of any other race/ethnic group (*p* < 0.001): about 30% indicated living in non-intact households before age 2 years and 19% between 2 and 6 years of age. Additionally, about 21% (10.5% before age 2 years and 10.5% after age 2 years) of all overweight/obese girls indicated living in non-intact households, compared with 14% (6.7% and 7.5%, respectively) for normal weight girls (*p* < 0.001). Girls from non-intact households lived in neighborhoods with lower median household incomes (*p* < 0.001) compared to their counterparts living in intact households. In breast development analyses, 10% of the girls were left-censored and 59% of girls were right-censored. Approximately 7% and 69% of girls were left- and right-censored for the pubic hair development analyses.

**Table 1:** Distribution of Characteristics by Household Intactness: KPNC Puberty Study (2009–2020), *N* = 26,044

Household intactness	Non-intact household before age 2 years (*n* = 2,034)		Non-intact household during age 2–6 years (*n* = 2,186)		Intact household (*n* = 21,824)		*P* value
	N	%	N	%	N	%	
Neighborhood Annual Household Income ($)^a, b^	65,088	(28,353)	68,670	(28,557)	77,833	(31,052)	< 0.001
Race/ethnicity							
White	533	5.6	757	8.0	8,187	86.4	< 0.001
Black	523	29.7	332	18.8	908	51.5	
Hispanic & Latinx	654	9.6	648	9.6	5,482	80.8	
Asian/Pacific Islander	208	3.6	286	4.9	5,364	91.6	
Other/Unknown	116	5.4	163	7.5	1,883	87.1	
Prepubertal BMI							
< 85th percentile	1,240	6.7	1,390	7.5	15,845	85.8	< 0.001
≥ 85th percentile	794	10.5	796	10.5	5,979	79.0	

*BMI* body mass index; *KPNC* Kaiser Permanente Northern California

^a^Values are expressed as mean (standard deviation)

^b^Calculated using 2010 Census data by address at or within one year of birth

### Primary analyses

#### Household intactness and thelarche

Girls who lived in a non-intact household before the age of 2 years and age 2–6 years had greater risk of experiencing earlier onset of thelarche compared with girls who lived in an intact household from birth until age 6 years (HR: 1.42, 95% confidence interval [CI]: 1.31,1.54; HR: 1.17, 95%CI: 1.08,1.26, respectively). After adjusting for race/ethnicity and median household income, associations were attenuated slightly but remained significant (HR: 1.29, 95%CI: 1.19,1.40; HR: 1.13, 95%CI: 1.04,1.22) (Table 2). 

**Table 2:** Association Between Household Intactness and Timing of Breast Development Onset: KPNC Puberty Study (2009–2020)

		Unadjusted				Adjusted^a^				Adjusted^a,b^			
Household Intactness	N	TR	95%CI	HR	95%CI	TR	95%CI	HR	95%CI	TR	95%CI	HR	95%CI
Non-intact household before age 2 years	1,915	0.96	0.96,0.97	1.42	1.31,1.54	0.97	0.97,0.98	1.29	1.19,1.40	0.98	0.97,0.99	1.25	1.14,1.36
Non-intact household during age 2–6 years	2,070	0.98	0.98,0.99	1.17	1.08,1.26	0.99	0.98,1.00	1.13	1.04,1.22	0.99	0.98,1.00	1.08	1.00,1.17
Intact household	20,765	Ref		Ref		Ref		Ref		Ref		Ref	

*CI* confidence interval; *HR* hazard ratio; *KPNC* Kaiser Permanente Northern California; *TR* time ratio

^a^Adjusted for race/ethnicity and median household income

^b^Adjusted for prepubertal BMI

#### Household intactness and pubarche

Girls who lived in non-intact households before age 2 and age 2–6 years were 48% (HR: 1.48, 95%CI: 1.35,1.61) and 26% (HR: 1.26, 95%CI: 1.16,1.38) more likely to experience earlier pubarche compared with girls who lived in intact households from birth until age 6 years, respectively. After adjusting for race/ethnicity and median household income, associations were attenuated but remained significant (HR: 1.21, 95%CI: 1.11,1.33; HR: 1.15, 95%CI: 1.05,1.25, respectively) (Table 3).

**Table 3:** Association Between Household Intactness and Timing of Pubic Hair Development Onset: KPNC Puberty Study (2009–2020)

		Unadjusted				Adjusted^a^				Adjusted^a,b^			
Household Intactness	N	TR	95%CI	HR	95%CI	TR	95%CI	HR	95%CI	TR	95%CI	HR	95%CI
Non-intact household before age 2 years	1,954	0.96	0.95,0.97	1.48	1.35,1.61	0.98	0.97,0.99	1.21	1.11,1.33	0.99	0.98,1.00	1.15	1.05,1.26
Non-intact household during age 2–6 years	2,115	0.98	0.97,0.99	1.26	1.16,1.38	0.99	0.98,0.99	1.15	1.05,1.25	0.99	0.98,1.00	1.11	1.02,1.21
Intact household	21,165	Ref		Ref		Ref		Ref		Ref		Ref	

*CI* confidence interval; *HR* hazard ratio; *KPNC* Kaiser Permanente Northern California; *TR* time ratio

^a^Adjusted for race/ethnicity and median household income

^b^Adjusted for prepubertal BMI

#### Household intactness and menarche

Girls who lived in non-intact households were more likely to experience early menarche (< 12 years old) compared to girls living in intact households. After adjusting for covariates, girls living in non-intact households before age 2 years had a 38% elevated risk (OR: 1.38, 95%CI: 1.17,1.62) and girls living in non-intact households ages 2–6 had 18% greater risk (OR: 1.18, 95%CI: 1.00,1.39) compared to girls who lived in intact households (Table 4).

**Table 4:** Association Between Household Intactness and Menarche: KPNC Puberty Study (2009-2020)

		Unadjusted		Adjusted^a^		Adjusted^a,b^		
Household Intactness	N	OR	95%CI	OR	95%CI	OR	95%CI	
Non-intact household before age 2 years	850	1.64	1.40,1.91	1.38	1.17,1.62	1.26	1.06,1.49	
Non-intact household during age 2–6 years	950	1.28	1.09,1.49	1.18	1.00,1.39	1.10	0.93,1.30	
Intact household	10,031	Ref		Ref		Ref		

*CI* confidence interval; *OR* odds ratio; *KPNC* Kaiser Permanente Northern California

^a^Adjusted for race/ethnicity and median household income

^b^Adjusted for prepubertal BMI

### Secondary Analyses

#### Mediating role of prepubertal BMI

Including girl’s prepubertal BMI did not substantially attenuate associations between exposure to non-intact household and timing of thelarche, pubarche, or menarche, and most associations remained statistically significant (Tables 2, 3 and 4).

#### Effect modification by race/ethnicity

The test for interaction by race/ethnicity in the association between household intactness and pubertal timing was significant in the thelarche (*p* < 0.001) and pubarche models (*p* = 0.02), but not in the menarche model (*p* = 0.94). We thus present race/ethnic stratified results for thelarche and pubarche (Tables 5-6). When stratified, the associations between living in non-intact households before age 2 and earlier thelarche were significant for White (HR: 1.24, 95%CI: 1.04,1.47), Black (HR: 1.60, 95%CI: 1.29,1.98) and Latinx (HR: 1.30, 95%CI: 1.12,1.51) girls. These associations approximate 2, 5, and 3 months earlier thelarche compared to those who lived in intact households, respectively. White (HR: 1.21, 95%CI: 1.06,1.38) and Black (HR: 1.44, 95%CI: 1.12,1.86) girls living in non-intact households at ages 2–6 also had increased risk of thelarche. There were no significant associations among Asian/Pacific Islanders.

**Table 5:** Association Between Household Intactness and Onset of Breast Development, Stratified by Race/Ethnicity: Test for Interaction, *p*<0.001

		Intact household	Non-intact household before age 2 years		Non-intact household during age 2–6 years	
Race/Ethnicity^a^	N	HR	HR	95%CI	HR	95%CI
White	9,084	Ref	1.24	1.04,1.47	1.21	1.06,1.38
Black	1,657	Ref	1.60	1.29,1.98	1.44	1.12,1.86
Hispanic & Latinx	6,411	Ref	1.30	1.12,1.51	1.00	0.85,1.16
Asian/Pacific Islander	5,542	Ref	1.05	0.81,1.37	0.85	0.68,1.07
Other/Unknown	2,056	Ref	1.35	0.95,1.91	1.69	1.26,2.26
Combined	24,750	Ref	1.29	1.19,1.40	1.13	1.04,1.22

*CI* confidence interval; *HR* hazard ratio

^a^Adjusted for median household income

Similar trends were observed in pubarche models. White girls living in non-intact households before age 2 years (HR: 1.35, 95%CI: 1.13,1.62), and Black girls living in non-intact households before age 2 years (HR: 1.42, 95%CI: 1.15,1.77) and age 2–6 years (HR: 1.39, 95%CI: 1.07,1.81) were more likely to experience earlier pubarche compared to their peers living in intact households, while no significant associations were found for Latinx or Asian/Pacific Islander girls.

**Table 6:** Association Between Household Intactness and Onset of Pubic Hair Development, Stratified by Race/Ethnicity: Test for Interaction, *p*=0.02

		Intact household	Non-intact household before age 2 years		Non-intact household during age 2–6 years	
Race/Ethnicity^a^	N	HR	HR	95%CI	HR	95%CI
White	9,170	Ref	1.35	1.13,1.62	1.15	0.99,1.32
Black	1,678	Ref	1.42	1.15,1.77	1.39	1.07,1.81
Hispanic & Latinx	6,572	Ref	1.05	0.90,1.23	1.08	0.93,1.27
Asian/Pacific Islander	5,714	Ref	0.94	0.68,1.31	0.96	0.74,1.24
Other/Unknown	2,100	Ref	1.45	1.00,2.09	1.44	1.06,1.96
Combined	25,234	Ref	1.21	1.11,1.33	1.15	1.05,1.25

*CI* confidence interval; *HR* hazard ratio

^a^Adjusted for median household income

## Discussion

To our knowledge, this is the largest prospective study of diverse adolescent girls to demonstrate that early-life exposure to family non-intactness is associated with earlier puberty. Girls who lived in a non-intact household before the age of 2 years were at greatest risk of experiencing earlier puberty compared with girls living in intact households. Later exposure (age 2–6 years) was also associated with early puberty, but not as strongly as exposure before age 2. Our study suggested that there may be racial/ethnic differences in these associations, where the associations were strongest among Black girls. Inclusion of prepubertal BMI did not substantially attenuate associations and, therefore, does not appear to be a likely mediator of these associations.

Our findings extend the previous knowledge regarding the associations between early-life family structures and pubertal timing in girls. Our results corroborate previous studies demonstrating that father absence is a risk factor of early puberty [9–15], as well as the studies that hypothesized that children may be particularly sensitive to stressful environments during the first few years of life [6]. There are several potential mechanisms explaining our observation.

First, infant attachment insecurity may be one mechanism through which girls living in non-intact households before age 2 years are experiencing earlier pubertal onset. Attachment security is the positive bond that develops between infant and caregiver that allows the infant to feel comfort while in the caregiver’s company [8]. Research shows that infants living in single-parent households are more likely to display attachment insecurity compared with infants living in dual-parent (e.g., intact) households [22]. Belsky and colleagues tested the hypothesis that girls with insecure attachment at 15 months would undergo earlier pubertal maturation in a cohort of 373 female infants who were followed over a period of 15 years [8]. The study found that girls with insecure infant attachment were more likely to experience earlier pubertal onset, earlier pubertal completion, and earlier menarche, compared with girls with secure infant attachment, lending evidence to the theory that family and rearing experiences are instrumental in determining pubertal and sexual developmental trajectories [8]. Our findings corroborate these observations.

Second, animal studies suggest there may be underlying biological mechanisms explaining the observed associations. For instance, a 2008 study found that maternal pup licking and grooming in rats correlates with estrogen receptor bioactivity, affecting female offspring pubertal timing [8, 23]. Female pups experiencing greater maternal care were less likely to engage in sexual activities and less likely to achieve pregnancy [8, 23]. It is possible that greater parental care in humans may also differentially affect the hypothalamic pituitary gonadal axis and other biological pathways involved in human sexual development. However, this theory is only plausible if the quality and amount of parental care varies substantially between single- and dual-parent households and is beyond the scope of the current study. In addition, there is a theory that pheromonal signaling may be one of the underlying mechanisms describing family intactness and pubertal timing. It has been postulated that proximity to the biological father may delay sexual development in girls as an evolutionary anti-inbreeding tactic to optimize quality of offspring [24]. Future studies further examining these mechanisms will provide greater insight.

The presence of race/ethnic differences in the association between household intactness and timing of pubertal onset may provide some explanation for why minority girls are experiencing an even greater decline in age of pubertal onset compared to other race/ethnic girls. In our race/ethnicity-stratified models, we found that White, Black, and Latinx girls, but not Asian/Pacific Islander or Other ethnicity girls, were at substantially greater risk of experiencing earlier breast onset, with Black and Latinx girls having the strongest associations. Although Black and Latinx have reportedly higher rates of childhood obesity and obesity-related conditions [25], our data did not demonstrate that the observed associations between family intactness and pubertal onset were explained by prepubertal BMI. Rate of single motherhood is disproportionately high in Black communities [16], which our data also demonstrated (i.e., nearly half of the girls living in a non-intact household by age 6). This, compounded by exposures to other stressors these communities often face (e.g., systemic racism), may partially explain the racial/ethnic differences in timing of puberty. Further, a previous study reported that socioeconomic status indicators such as mother’s marital status and lower family income accounted for up to 50% of the increased risk of earlier menarche found in Black and Latinx girls [26]. Other individual-level factors such as socioeconomic status, perceived stress, or adverse childhood events may further explain the differences [27].

Lastly, emerging research demonstrates that social and built environments are strong predictors of health outcomes independent of individual-level factors [27–32]. Living on a single income in the San Francisco Bay Area, a region with notoriously high cost of living, may pressure single parents to live in less optimal neighborhoods than intact families that are likely to have higher household incomes. Further, neighborhood characteristics may partially explain race/ethnic differences in pubertal timing [33]. We controlled for neighborhood-level household income at birth but did not consider changes in address, and therefore changes in neighborhood quality that could have occurred during the girl’s childhood, especially following family dissolution. Additionally, our measure of neighborhood-level household income was based on 2010 Census data and thus represents a single point in time. It may not accurately reflect changes in neighborhoods resulting from housing development, gentrification, and other factors that may be independent sources of stress. Future studies that include these multi-level factors and their changes over time will shed light on the contexts in which household intactness is associated with pubertal timing, and how these factors may influence associations by race/ethnicity. Such knowledge will help elucidate the complex interplay of race/ethnicity, socioeconomic status, neighborhood, stress and child development. This will in turn help the design of upstream interventions to slow the hastening of onset of pubertal development and race/ethnic health disparities due to earlier pubertal timing in minority populations.

This study has several limitations. Lack of detailed information on family structure prevented us from distinguishing single mother vs. single father households, presence of same-sex parenting, presence of other adult caregivers in the home, number and ages of siblings or other household members, and other circumstances pertaining to child guardianship including foster care status. Having a more robust understanding of a child’s living arrangements would permit us to explore in more detail the nuances of family structure and parent involvement on pubertal maturation. Second, because this study was based on EHR data, we did not have information on other potential confounders such as mother’s age at menarche, individual-level household income, perceived stress, food and nutrition intake, and physical activity. Last, we used EHR-based Tanner Stages assessed primarily by pediatricians. Although these data are far better than parent- or child-reported Tanner Stages, they may not be as precise as assessments by trained endocrinologists. However, these limitations of the study are outweighed by numerous strengths. Because of the availability of the EHR data, we were able to conduct one of the largest prospective cohort studies with availability of objective measures of clinical outcomes (e.g., Tanner Stages, BMI), and other relevant confounder variables, providing an unparalleled opportunity to rigorously and efficiently examine the association of intact household status on onset of puberty. Other notable strengths of the study include a diverse cohort with substantial representation of race/ethnic groups, lack of which has been a major limitation in previous studies.

## Conclusions

Early life exposure to household non-intactness is associated with early pubertal timing in girls, and this association may vary by race/ethnicity. Although conditions of home and family life cannot be modified in a clinical setting, it is important for pediatric specialists to understand the potential adverse associations between early-life family-related factors and a girl’s pubertal trajectory and to identify girls who may be at increased risk for early puberty. This knowledge can facilitate conversations with the caregivers of young girls, who can work with providers to pinpoint areas of home life that can be improved through social and family services.

## Data Availability

The datasets generated and/or analyzed during the current study are not publicly available due to our institutional policy. Individuals who are interested in accessing the data may contact the corresponding author regarding [or to discuss or set up] a data use agreement.

## Abbreviations

BMI: Body Mass Index
CDC: Centers for Disease Control and Prevention
CI: Confidence Interval
CYGNET: Cohort Study of Young Girls' Nutrition, Environment, and Transitions
EHR: Electronic Health Record
HR: Hazard Ratio
KPNC: Kaiser Permanente Northern California
OR: Odds Ratio
SAS: Statistical Analysis System
TR: Time Ratio
